# The α_1_-adrenoceptor inhibitor ρ-TIA facilitates net hunting in piscivorous *Conus tulipa*

**DOI:** 10.1038/s41598-019-54186-y

**Published:** 2019-11-28

**Authors:** Mriga Dutt, Jean Giacomotto, Lotten Ragnarsson, Åsa Andersson, Andreas Brust, Zoltan Dekan, Paul F. Alewood, Richard J. Lewis

**Affiliations:** 1 Centre for Pain Research, Institute for Molecular Bioscience, The University of Queensland, St. Lucia, QLD 4072 Australia; 2 Queensland Brain Institute, The University of Queensland, St. Lucia, QLD 4072 Australia; 3 Queensland Centre for Mental Health Research, West Moreton Hospital and Health Service and University of Queensland, Brisbane, Australia

**Keywords:** Mechanism of action, Peptides

## Abstract

Cone snails use separately evolved venoms for prey capture and defence. While most use a harpoon for prey capture, the Gastridium clade that includes the well-studied *Conus geographus* and *Conus tulipa*, have developed a net hunting strategy to catch fish. This unique feeding behaviour requires secretion of “nirvana cabal” peptides to dampen the escape response of targeted fish allowing for their capture directly by mouth. However, the active components of the nirvana cabal remain poorly defined. In this study, we evaluated the behavioural effects of likely nirvana cabal peptides on the teleost model, *Danio rerio* (zebrafish). Surprisingly, the conantokins (NMDA receptor antagonists) and/or conopressins (vasopressin receptor agonists and antagonists) found in *C*. *geographus* and *C*. *tulipa* venom failed to produce a nirvana cabal-like effect in zebrafish. In contrast, low concentrations of the non-competitive adrenoceptor antagonist ρ-TIA found in *C*. *tulipa* venom (EC_50_ = 190 nM) dramatically reduced the escape response of zebrafish larvae when added directly to aquarium water. ρ-TIA inhibited the zebrafish α_1_-adrenoceptor, confirming ρ-TIA has the potential to reverse the known stimulating effects of norepinephrine on fish behaviour. ρ-TIA may act alone and not as part of a cabal, since it did not synergise with conopressins and/or conantokins. This study highlights the importance of using ecologically relevant animal behaviour models to decipher the complex neurobiology underlying the prey capture and defensive strategies of cone snails.

## Introduction

Cone snails are a group of ~800 species of marine molluscs endemic to Indo-Pacific and Atlantic coral reef systems^1^. They possess a long venom duct that can differentially secrete compartmentalised predatory and defensive venoms^2^. These molluscs prey on polychaete annelids (vermivores), molluscs (molluscivores) or fish (piscivores) using venom peptides injected intramuscularly (i.m.) through a hollow radula tooth harpooned into prey^3^. The injected venom of an individual cone snail contains >1000 cysteine-rich peptides (conopeptides or conotoxins) that target a diverse range of neuronal ion channels and cell membrane receptors^4,5^. Piscivorous cone snails mostly use a harpoon strategy^3,6^ that deploys excitatory (lightning-strike cabal) or inhibitory (motor cabal) conotoxins to paralyse fish^7–9^. Additionally, the Gastridium clade, including the well-studied *Conus geographus* and *Conus tulipa*, use a net hunting strategy where so called “nirvana cabal” peptides are secreted into the water to inhibit the escape response of teleost fish^7^, allowing capture of large fish (see Supplementary Movie 1 of ref. ^2^). Early studies hypothesised the nirvana cabal comprised non-paralytic peptides including conantokins and conopressins^10,11^. However, any potential involvement in the nirvana cabal was extrapolated from their identified physiological effects following their intracerebral injection in rodents^11^ and direct effects of most postulated nirvana cabal peptides on fish when added directly to aquaria water have not been shown.

Venomic studies of *C*. *geographus* revealed the presence of non-paralytic conantokins, conopressins, contulakins and conoinsulins in the distal duct segment where the predatory-evoked venoms are secreted in this species^2,12–14^. Recent venomic studies of *C*. *tulipa* support a similar distribution, with conantokins dominant in the distal duct section, the α_1_-adrenoceptor (α_1_-AR) antagonist ρ-TIA and vasopressin receptor (VR) antagonist conopressin-T dominant in the proximal central duct, whereas conoinsulins were only detected at low levels in the *C*. *tulipa* transcriptome^15^. ρ-TIA is an allosteric antagonist of the mammalian α_1_-AR that binds to a well-characterized pharmacophore on the extracellular surface of this family A GPCR^16,17^. While injection of ρ-TIA into fish did not reveal a phenotype^16^, previous studies examining the effects of prazosin on fish suggest ρ-TIA might also induce a sleep-like state in fish^18^. However, with the exception of the conoinsulins^19^, the behavioural effects of these potential nirvana peptides added to aquarium water housing fish has not been established to confirm their potential role in net hunting.

In this study, we analysed behavioural effects on the teleost zebrafish induced by synthetic candidate nirvana cabal conotoxins added to their surrounding water to characterize their potential to contribute to net hunting. Zebrafish larvae behaviour was monitored using an automatic tracking system (Zebrabox Revolution) that allows real-time measurement of swim speed, scoot distance and angle of turn behaviours^20–27^. Surprisingly, conopressins and conantokins had no detectable effect on the fish ‘fight or flight’ response either alone or in combination, whereas ρ-TIA potently blocked the zebrafish escape response to mechanical touch stimuli. N-terminal truncations of ρ-TIA and site-directed mutagenesis of the zebrafish α_1_-AR confirmed that ρ-TIA acted at a related allosteric site in the zebrafish α_1_-AR. This study directly implicates ρ-TIA as an antagonist at the zebrafish α_1_-AR that may contribute to the nirvana cabal, allowing fish capture directly by mouth without harpooning.

## Results

### Systemic effects of dissected *C*. *tulipa* venom in adult zebrafish

**Figure 1:**
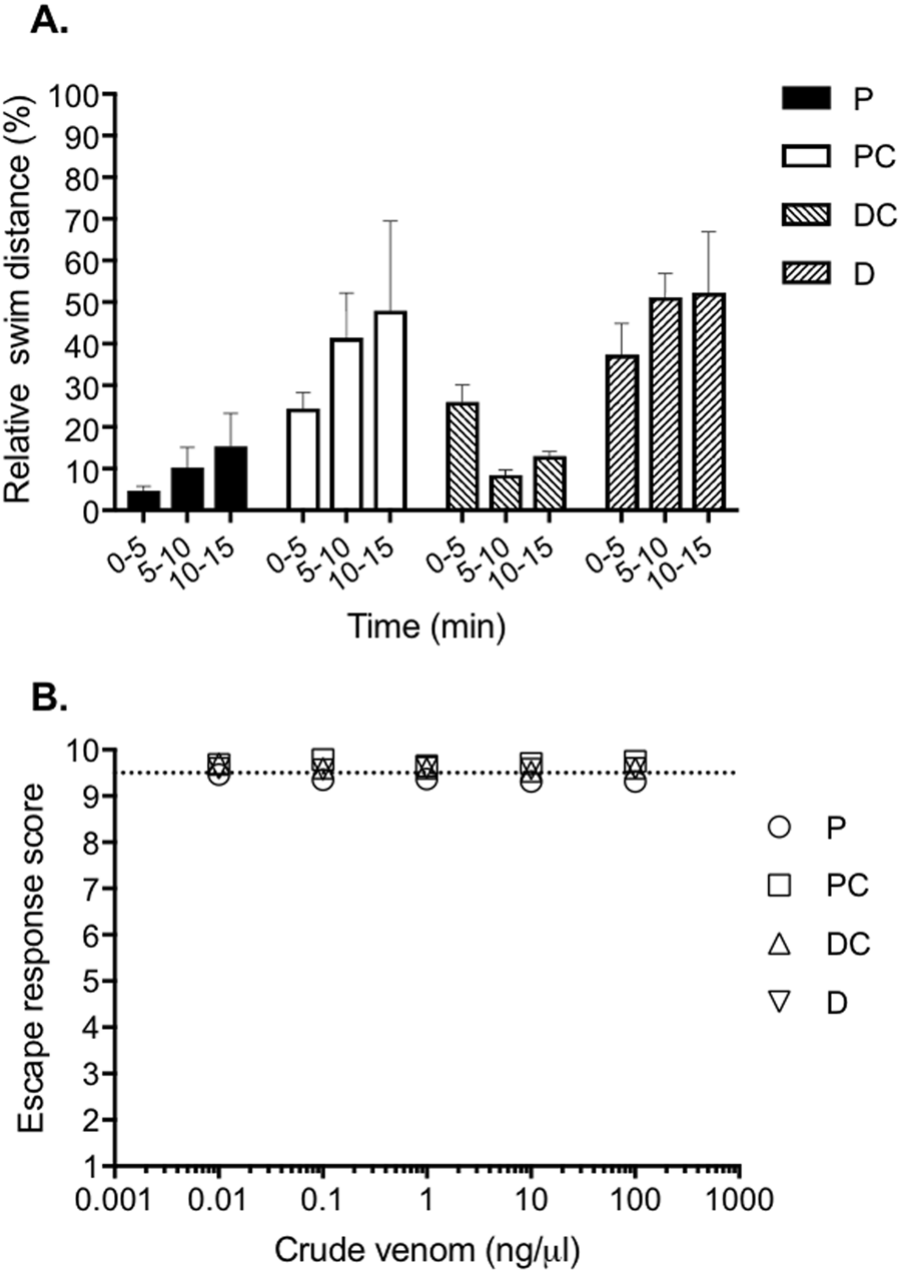
Phenotypic effects of dissected venom on zebrafish. (**A)** Effects of intramuscular dissected *C*. *tulipa* venom from the proximal (P), proximal central (PC), distal central (DC) and distal (D) duct sections in adult zebrafish (0.1 μg i.m.) on swim distance was recorded for 15 min relative to the swim distance of untreated fish. **(B**) Dose dependent effects of *C*. *tulipa* crude venoms (0.001‒100 ng/μl) dosed in water on touch-evoked escape responses of 5-dpf zebrafish larvae. Untreated larvae showed an average escape response score of 9.5 (dotted line). For both experiments, data are expressed as the mean ± SEM obtained of six independent experiments.

To determine the venom duct localisation of peptides contributing to harpoon prey capture, we extracted *C*. *tulipa* dissected venom from four duct sections, proximal (P), proximal central (PC), distal central (DC) and distal (D), and administered 0.1 µg of each intramuscularly (i.m.) and monitored for any behavioural changes. Venom from each of the duct sections reduced the swimming ability of fish, with the P section being most potent and causing an 80% reduction in total swim distance relative to the control (Fig. 1A). Dissected venom from each section produced flaccid paralysis that drastically slowed swimming movements, consistent with a motor cabal effect. Fish injected i.m. with the P dissected venom displayed an immediate and almost complete loss of motor activity that was irreversible over 15 min. In contrast, fish administered with the DC dissected venom had a delayed onset of activity, while PC and D had similar but weaker effects, reducing the total swim distance by 50% relative to the control.

### Dissected *C*. *tulipa* venom did not induce a nirvana cabal effect in larval zebrafish

In an attempt to establish the duct localisation of nirvana cabal peptides, we administered dissected venoms to the fish water column and monitored for any change in fish behaviour using 5-day post fertilisation (5-dpf) zebrafish larvae in multi-well assay plates. Addition of dissected *C*. *tulipa* duct venom (0.001‒100 ng/µl) from each of the duct sections failed to alter the fish escape response (Fig. 1B) and all larvae displayed normal swimming behaviour.

### Conantokin and conopressin have no effect on fish escape responses

**Figure 2:**
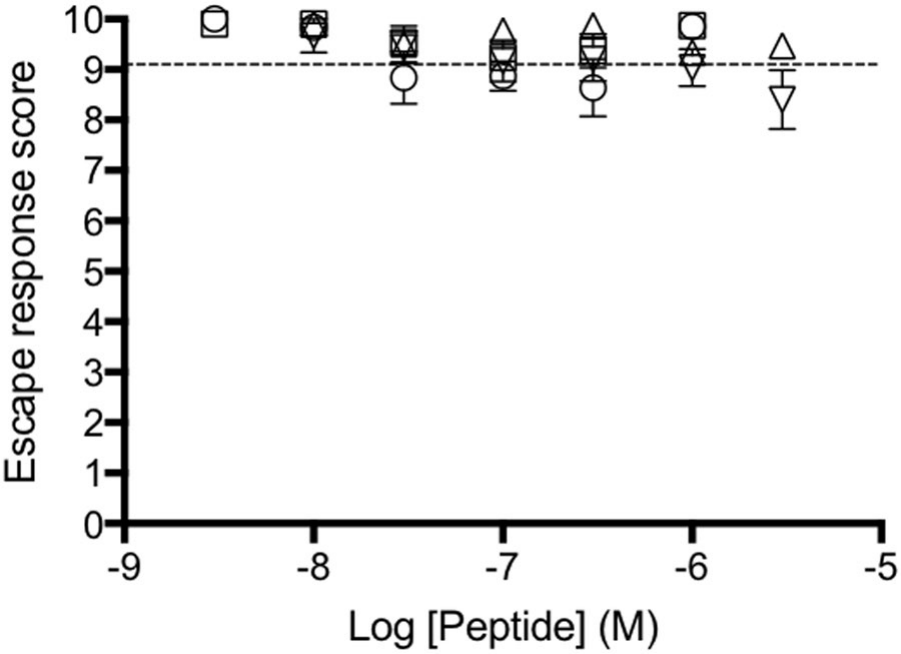
Synthetic conantokins and conopressins are ineffective on the zebrafish larval escape response. The four nirvana peptides, conantokin-G (circle), conantokin-T (square) (0.003–1 μM), conopressin-G (inverted triangle) and conopressin-T (triangle) (0.003–5 μM) were dosed in water and touch-evoked larval escape responses plotted. All four peptides at maximal concentrations failed to elicit any effect on the larvae, as they constantly displayed an escape response score of around 9. Untreated larvae exhibited an average escape response score of 9.2 (dotted line). Data are expressed as the mean ± SEM of three independent experiments.

Administration of dissected venom to larval water failed to produce nirvana cabal-like activity in zebrafish larvae, perhaps because the relevant peptides are secreted only on demand. We thus decided to test the hypothesised synthetic nirvana peptides individually and in combination on larval zebrafish. Neither the VR antagonist conopressin-T nor the NMDAR antagonist conantokin-T from *C*. *tulipa* affected the larval escape response at concentrations up to 1 µM (Fig. 2). Similarly, neither the VR agonist conopressin-G nor conantokin-G from *C*. *geographus* affected the larval escape response at concentrations up to 5 µM (Fig. 2). In all treatment groups, exposed larvae displayed normal swimming behaviour indistinguishable from the control group.

### ρ-TIA reduces the fish escape response in larval zebrafish

**Figure 3:**
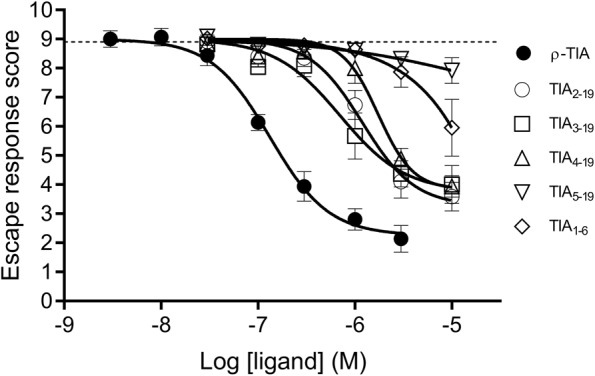
ρ-TIA induced escape response on zebrafish larvae. The potential of ρ-TIA and its analogues (0.003–10 µM) to affect the escape response of treated zebrafish larvae, when presented with a light mechanical stimulus, was analysed using the manual touch-evoked assay. Larvae treated with ρ-TIA displayed a loss of escape response in a concentration dependent manner, where maximal ρ-TIA concentration (10 µM) completely deprived the larvae of their escape abilities. Sequential truncation of the N-terminal residues of ρ-TIA produced analogues (TIA_2–19_, TIA_3–19_, TIA _4–19_) that induced a reduction in escape responses to the mechanical touch stimulus, with lower potencies compared to ρ-TIA. Absence of the N-terminal sequence “FNWR” from the peptide (TIA_5–19_) and the amino acid sequence “FNWRCC” alone (TIA_1–6_) had no significant effect on the larvae flight response at up to 10 µM. Larvae exposed to negative control (E3) exhibited normal escape response behaviour, with an average score of 8.9 (dotted line). Data is expressed as ± SEM and is the mean of values obtained from six independent experiments, each consisting of ten individual touch-evoked scores.

Following a preliminary screen of synthetic conotoxins, we identified that ρ-TIA (0.03‒10 µM) added to the water column produced a striking loss of zebrafish larvae escape response to light mechanical touch on their trunk or tail. This effect was dose-dependent, with an EC_50_ of 0.19 ± 0.62 µM. ρ-TIA at concentrations above 3 µM produced an acute reduction in swimming speed, with the larvae exhibiting an apparent rigid body with minimal gill movements and abnormal escape responses (escape response score 2–3 vs. a control score of 8.9 of 10). At 1 µM, reduced escape responses developed over the initial 5 min of exposure to ρ-TIA, allowing larvae to be easily pushed around with the Teflon prod without showing escape or avoidance responses. (Fig. 3, Supplementary Video). The effects of 1 μM ρ-TIA lasted beyond the 15 min observation period, while prolonged exposure to ρ-TIA (>3 μM) was lethal to larvae. Additionally, ρ-TIA induced bursts of erratic swimming, similar to a stress response but with a stronger swimming response (Supplementary Fig S1). This altered swimming behaviour was observed for ~1 min after addition of ρ-TIA at concentrations above 0.01 μM, and was most pronounced at 0.3 µM ρ-TIA.

### Assessing synergistic effects of ρ-TIA with presumed nirvana cabal conotoxins

**Figure 4:**
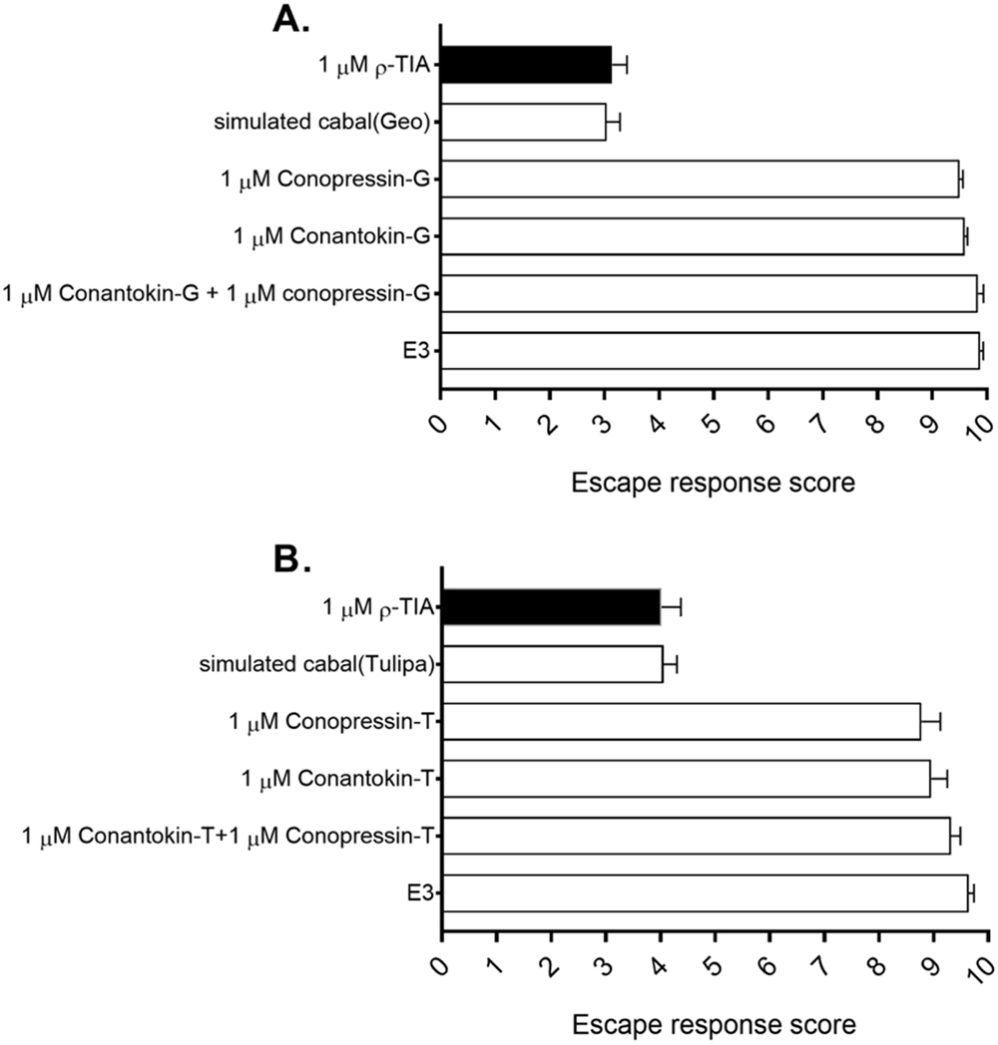
Simulation of the nirvana cabal on zebrafish larvae. The prey capture environment was simulated on the zebrafish larvae using mixtures of the nirvana peptides. Equimolar concentrations (1 μM) of synthetic conantokins and conopressins from **(A)** *C*. *geographus* and **(B)** *C*. *tulipa* were applied to the zebrafish larvae to observe for effects on their sensory capabilities. In both experiments, co-addition of conopressins and conantokins did not induce any synergistic effect on the escape response of the larvae, whereas a reduction in the larval flight response was observed when 1 μM ρ-TIA was added to the mixture (simulated Geo/Tulipa cabal). The simulated cabal from *C*. *geographus* peptides produced an average escape response score of 3.06, while the *C*. *tulipa* peptides produced an average escape response score of 4.07. In both experimental conditions, this observed effect was parallel to that elicited by single addition of 1 μM ρ-TIA (black solid bar), thus demonstrating that the hypothesised nirvana peptides do not affect the sensory capabilities of larval zebrafish. Single additions of the conopressins, conantokins and E3 (negative control) were ineffective on larval escape behaviour. Data is expressed as ± SEM and is the mean of values obtained from five independent experiments, each consisting of ten individual touch-evoked scores.

To determine if the observed nirvana cabal effect results from synergy between different peptide classes, we treated the larvae with different combinations of conopressins, conantokins and ρ-TIA, each dosed at 1 µM. Co-exposure of zebrafish to conopressins and conantokins from *C*. *geographus* (Fig. 4A) or *C*. *tulipa* (Fig. 4B) had no effect on larval zebrafish escape responses, and exposed zebrafish instantly displayed the characteristic “C-start”^28^ response when presented with the mechanical stimulus. The escape responses for ρ-TIA in the presence of the conantokin/conopressin combinations were also indistinguishable from the responses observed for ρ-TIA alone (Fig. 4).

### ρ-TIA N-terminal tail is critical for the induced phenotypic effect

Sequential deletion of the four N-terminal residues (TIA_2–19_, TIA_3–19_ and TIA_4–19_) reduced ρ-TIA potency to inhibit the zebrafish escape response (Fig. 3, Table 1), with deletion of the entire N-terminal tail FNWR (TIA_5–19_) abolishing inhibition of the escape response (Fig. 3).

**Table 1:** N-terminal truncated analogues of ρ-TIA. The amino acid sequence, disulfide connectivity and IC_50_ ± SEM to inhibit zebrafish escape response.

Peptide	Amino acid sequence	Disulfide connectivity	IC_50_ (μM)
ρ-TIA	FNWRCCLIPACRRNHKKFC*	1–3, 2–4	0.19 ± 0.62
TIA_2–19_	NWRCCLIPACRRNHKKFC*	1–3, 2–4	1.34 ± 0.18
TIA_3–19_	WRCCLIPACRRNHKKFC*	1–3, 2–4	4.09 ± 1.12
TIA_4–19_	RCCLIPACRRNHKKFC*	1–3, 2–4	3.16 ± 1.01
TIA_5–19_	CCLIPACRRNHKKFC*	1–3, 2–4	>10
TIA_1–6_	FNWRCC*	1–2	>10

*C-terminal amidation.

### ρ-TIA affinity at zebrafish α_1B_ adrenergic receptors (ZF α_1BB-_AR)

**Figure 5:**
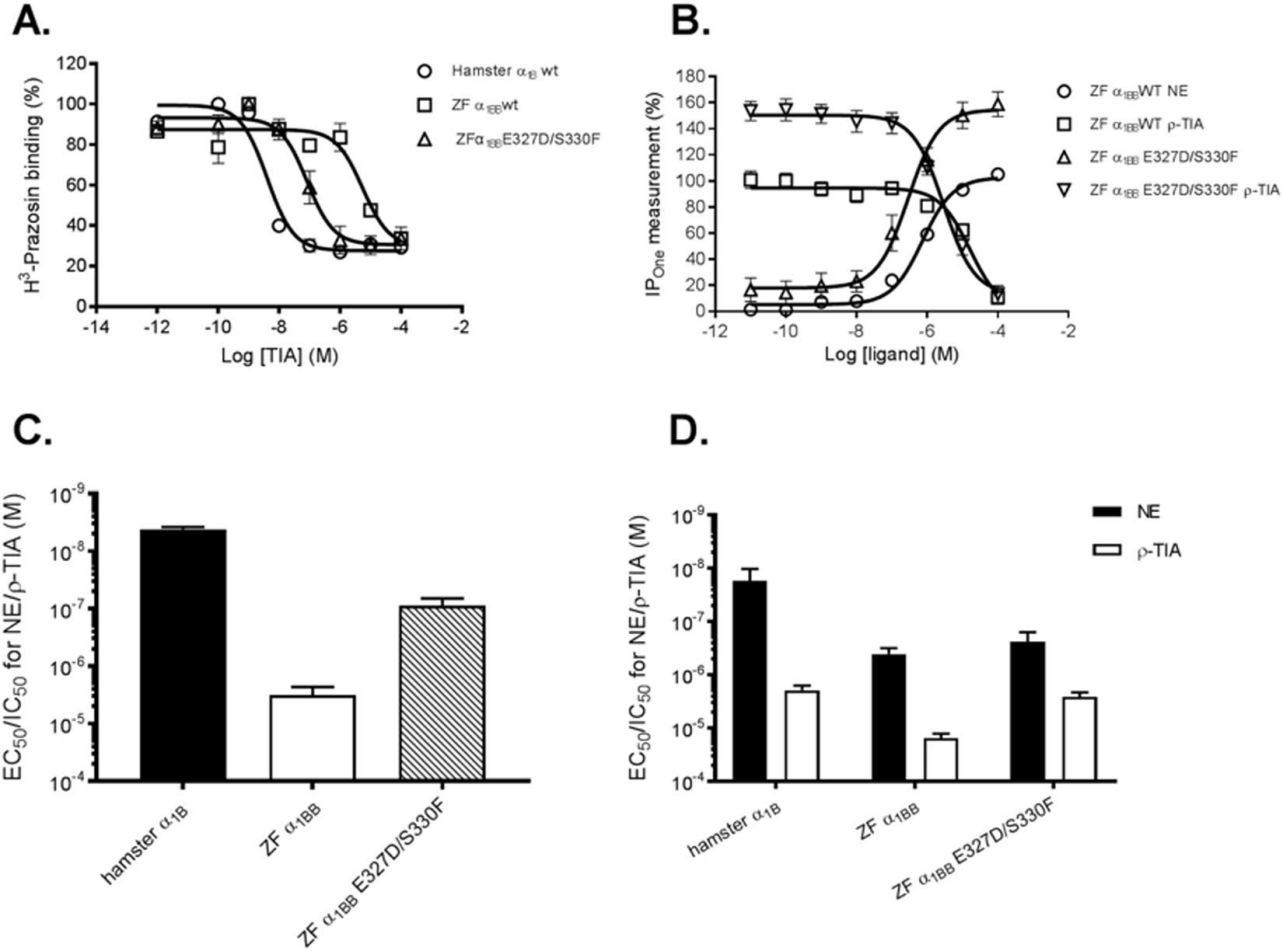
ρ-TIA is a functional antagonist of the zebrafish α_1_-AR. (**A**,**C**) ρ-TIA displayed weak binding affinity (IC_50_ = 3198.9 ± 46.26 nM) to the zebrafish wild type α_1BB_-AR, while a 36-fold improvement in binding affinity was observed at the zebrafish α_1BB_-E327D/S330F double mutant (IC_50_ = 88.51 ± 11.14 nM). (**B**,**D**) Inhibition of NE induced IP_1_ accumulation in cells expressing the ZFα_1BB_-E327D/S330F-AR by ρ-TIA was determined using the IP-one HTRF assay. A 6-fold improvement in ρ-TIA potency was observed at the double mutant (IC_50_ = 2.61 ± 0.22 µM) when compared to the zebrafish WTα_1BB_-AR (IC_50_ = 15.26 ± 1.19 µM). Data has been expressed as ± SEM and is presented as an average of three to six independent experiments, each performed in triplicate.

ρ-TIA affinity at WT and mutant ZF α_1BB_-AR was determined using a ^3^H-prazosin radioligand binding assay (Fig. 5A,C). E327D/S330F-ZFα_1BB_-AR, mutated to introduce the ρ-TIA pharmacophore in mammalian α_1B-_ARs (Table 2, Supplementary Fig. S2) had 36-fold enhanced affinity for ρ-TIA (IC_50_ = 88.51 ± 11.14 nM) compared with WT ZFα_1BB_-AR (Fig. 5A, Table 3). ρ-TIA was a partial inhibitor of prazosin binding at the zebrafish α_1BB_-AR (Fig. 5A), as previously reported at hamster α_1B_-AR^17^.

**Table 2:** ρ-TIA pharmacophore residues in hamster α_1_-AR^17^ and corresponding residues in human and zebrafish α1-ARs.

Receptor	Residues influencing ρ-TIA binding				
Hamster α_1B_	D327	F330	V197	S318	E186
Human α_1B_	D326	F329	V196	S317	E185
ZF α_1AA_	D318	F321	I192	S309	E181
ZF α_1AB_^*^	D317	F320	V188	S308	E177
ZF α_1BA_	E320	F323	I199	S311	E183
ZF α_1BB_^*^	E327	S330	I194	S318	E188

*Sequence confirmed from NCBI database.

**Table 3:** ρ-TIA potency at hamster and zebrafish α_1_-adrenoceptors. IC_50_ ± SEM values (n = 3–6) are shown.

Receptor	Radioligand binding assay (nM)	IP-one functional assay (μM)
Hamster α_1B_	4.39 ± 0.14 (3)	1.56 ± 0.17 (3)
Zebrafish α_1BB_	3198.9 ± 46.26 (3)	15.26 ± 1.19 (6)
Zebrafish α_1BB_ E327D/S330F	88.51 ± 11.14 (3)	2.61 ± 0.22 (4)

### ρ-TIA is a functional antagonist of the ZF α_1_-AR

NE-activated signalling to inositol 1-phosphate (IP_1_) was not significantly different for ZF WT (EC_50_ = 407.17 ± 45 nM) and E327D/S330F mutant (EC_50_ = 238.92 ± 4.28 nM) (Fig. 5B,D). ρ-TIA inhibited 10 µM NE-stimulated accumulation of IP_1_ at the E327D/S330F mutant (IC_50_ = 2.61 ± 0.22 μM) with 6-fold enhanced potency compared with the WT ZF receptor (Table 3).

## Discussion

The venom of piscivorous snails has been extensively studied for its pharmacological potential and more recently to understand the evolution of their predatory and defensive strategies. The Gastridium clade, including the well-studied *C*. *geographus* and *C*. *tulipa*, have developed a net hunting strategy to catch teleost fish directly by mouth using venom secreted directly into the surrounding water. However, the conotoxins contributing to this unique net hunting strategy remain unclear. In this study, we developed a zebrafish larval assay using an automated screening platform to quantify behavioural changes. Surprisingly, the dissected *C*. *tulipa* venom from each of the four duct sections added to the water column had no effect on the zebrafish escape response, suggesting nirvana cabal peptides are normally present in the venom duct at low concentrations and presumably upregulated and secreted into the water during net hunting. In contrast, i.m. injection of these dissected venoms produced flaccid paralysis in zebrafish that dramatically reduced their ability to swim, especially the proximal duct venom where defensive venom peptides are produced in the closely related *C*. *geographus*^2^, supporting their putative role in defence in *C*. *tulipa*.

Previous studies have suggested conantokins, conopressins, contulakins and conoinsulins as the putative nirvana peptides^11,19,29^. However, apart from the conoinsulins, the putative role of these non-paralytic conotoxins is based on their effects on rodents. In the search for putative nirvana cabal peptides, we used the zebrafish research model to assess the potential of conopressins and conantokins, commonly found in *C*. *geographus* and *C*. *tulipa* venom, to contribute to the nirvana cabal. Contrary to expectation, conopressins and conantokins previously identified from *C*. *geographus* and *C*. *tulipa*, administered either alone or in combination to aquarium water, had no significant effect on zebrafish escape responses and overall behaviour. In contrast, the α_1_-AR receptor antagonist ρ-TIA added to aquarium water dramatically reduced the ability of the zebrafish to respond to mechanical stimuli. ρ-TIA was previously found to be non-lethal to fish by injection^16^ and was prominent in the central but not the proximal duct section^15^, suggesting it does not contribute to the defence-evoked venom of *C*. *tulipa*. Co-addition of conopressins and conantokins together with ρ-TIA failed to alter the ρ-TIA effect, suggesting that conopressins and conantokins probably do not contribute to a nirvana cabal in *C*. *tulipa* and raising the possibility that ρ-TIA may act alone to facilitate net feeding in this species. It remains to be determined if the conopressins and conantokins contribute to the nirvana cabal in *C*. *geographus* together with the contulakins and conoinsulins commonly found in this species.

ρ-TIA has previously been shown to be specific for α_1_-ARs over ligand and voltage-gated ion channels in nerves and skeletal muscle^16^. To identify the target of ρ-TIA in zebrafish, we assessed the ability of N-terminal truncations of ρ-TIA, previously identified as critical for allosteric inhibition at mammalian α_1_-ARs^30^, to inhibit the zebrafish escape response. In parallel with reductions observed in mammalian ARs, these analogues had a reduced potency to inhibit the escape response, consistent with inhibition of fish α_1_-ARs being the target for the observed sensory deprivation, and suggesting that ρ-TIA acts similarly on mammalian and zebrafish α_1_-ARs. ρ-TIA did not fully inhibit ^3^H-prazosin binding to zebrafish α_1_-ARs, consistent with the non-competitive effect seen for mammalian α_1_-ARs^31^. The introduction of mammalian α_1_-AR pharmacophore residues previously identified as important for ρ-TIA binding^17^ into the zebrafish AR (E327D/S330F-ZFα_1BB_-AR) enhanced ρ-TIA affinity 36-fold. Together, these data indicate that ρ-TIA can act at low nanomolar concentrations and likely binds at the same allosteric site on mammalian and zebrafish α_1_-ARs. Interestingly, both of the mutated pharmacophore residues are found in ZFα_1AA_- and ZFα_1AB_-ARs (Table 2), while F330 is found in ZFα_1BA_-AR, suggesting ρ-TIA may be a useful probe to help identify which of the four zebrafish α_1_-ARs contribute to this loss of escape response observed.

In addition to inhibiting the escape response of fish, ρ-TIA treated larvae also showed erratic bursts of swimming, reminiscent of the hyperactivity described for excitatory κA-conotoxins in adult zebrafish^32^. However, the excitatory effects observed with ρ-TIA lasted ~1 min and were fully reversible, with larvae resuming normal swimming after the hyperactive state, in contrast to the irreversible paralysis produced by κA-conotoxins. Neurotransmitter function is an important regulator of locomotion in adult fish, and disruption by antipsychotic drugs such as fluphenazine and haloperidol produces similar erratic swimming motion in zebrafish^33^. It is possible that upon initial exposure, ρ-TIA may temporarily interfere with the lateral line sensory neurons, disrupting normal function.

Through a combination of behavioural and pharmacological assays on zebrafish, we have identified that ρ-TIA present in *C*. *tulipa* venom retains its antagonistic properties towards the fish α_1_-ARs and reverses the stimulatory effects of norepinephrine on fish to induce a “sleep-like” quiescent state^18^. This observed biological effect appears to be parallel to the sensory deprivation effect induced during net predation, which might facilitate fish capture by mouth by these nocturnal feeders. Thus, we hypothesise that ρ-TIA plays a direct biological role in net hunting by *C*. *tulipa*. The low levels of ρ-TIA identified in the venom duct suggest that secretion of ρ-TIA is stimulated only when *C*. *tulipa* starts net hunting. In contrast, conopressins and conantokins appear to play little role in the net hunting strategy of *C*. *tulipa*, raising questions about their biological function in this venom. Conantokins in particular have a dominant distal venom expression^13,15^ and yet there is no definitive study implicating their potential role in prey capture and/or defence. Given conantokins are expressed in several piscivorous venoms^34–37^, their involvement in the hook and line prey capture strategy warrants further investigation. Similarly, the inactivity of conopressin-T in the behavioural assays further supports its potential role in the defensive venom of *C*. *tulipa*^15^. Although ρ-TIA may act alone to facilitate net hunting in *C*. *tulipa*, we cannot exclude the possibility that other conotoxins, yet to be identified, may act in synergy. In contrast, net hunting in *C*. *geographus* may be facilitated by conoinsulins, which alone have been shown to affect fish behaviour at 25 µM^19^, and/or contulakins, which are both prominent in *C*. *geographus* but not *C*. *tulipa* venom. Finally, development of a milking strategy for the net hunters of the Gastridium clade that allows direct isolation and sequencing of the “nirvana cabal” proteome is required to further characterise the peptides responsible for this unique predatory strategy that profoundly alters fish behaviour. Such peptides are expected to provide new research tools that are useful in the elucidation of the neurobiology of escape responses and sleep in fish.

## Methods

### Zebrafish husbandry

Adult and embryos zebrafish (TU strain) were maintained in accordance with the standards and guidelines approved by the University of Queensland Animal Ethics Committee (UQ AEC; AEC approval number IMB/158/15/NHMRC). Embryos were collected through mating pairs in specialised mating tanks (Tecniplast, USA), maintained at 28°C in E3 embryo water (2.9 g NaCl, 0.13 g KCl, 0.48 g CaCl_2_, 0.82 g MgSO_4_.7H_2_O, 0.01% v/v methylene blue in 1 L MilliQ water). For the larvae behaviour assays, healthy 5-dpf animals were selected and distributed into 24-multi-well plates (Corning^®,^ USA) supplemented with 500 µl of E3 medium. All described zebrafish behaviour assays were designed and performed according to the animal ethics guidelines approved by the UQ AEC (AEC approval numbers IMB/158/15/NHMRC and IMB/130/15/NHMRC).

### Venom peptides

A single specimen of *C*. *tulipa* was sacrificed and the venom duct was dissected. The duct was divided into four equal parts; proximal (P), proximal central (PC), distal central (DC) and distal (D) section and dissected venom extracted separately from each section, as described previously^38^. Conantokin-G, conantokin-T, ρ**-**TIA and its N-terminal truncated analogues were chemically synthesized as described below. All peptides were made up to the required stock concentrations in E3 medium and stored at 4°C prior to use.

### Peptide synthesis

Peptides were assembled by SPPS on a Symphony automated peptide synthesizer (Protein Technologies Inc., Tucson, AZ, USA) by Fmoc chemistry on 0.1 mmol scale and Fmoc-Rink-amide polystyrene resin (substitution value 0.67 mmol/g). Fmoc deprotection was accomplished by treatment with 30% piperidine/DMF (1 × 1 min, then 1 × 3 min). Amino acids were coupled using five equivalents of Fmoc amino acid/HCTU/DIEA (1:1:1) relative to resin loading (1 × 4 min then 1 × 8 min). Side-chain protecting groups used were as follows: Arg(Pbf), Asn(Trt), Asp(tBu), Cys(Trt or Acm), Gla(tBu_2_), Gln(Trt), Glu(tBu), His(Trt), Lys(Boc), Ser(tBu), Thr(tBu), Trp(Boc) and Tyr(tBu). Cleavage from the resin and removal of side-chain protecting groups was achieved by treatment with 95% TFA/2.5% TIPS/2.5% H_2_O for 2 h at room temperature. The crude product was precipitated with cold diethyl ether (Et_2_O), washed with Et_2_O, redissolved in 50% acetonitrile/0.1% TFA/H_2_O and lyophilised. RP-HPLC solvent A was 0.05% TFA/H_2_O and solvent B was 0.043% TFA/90% acetonitrile/H_2_O. Analytical HPLC was performed on a Shimadzu LC20AT system using a Thermo Hypersil GOLD C18 2.1 × 100 mm column and a gradient of 0–40% B over 40 min at flow rate of 0.3 mL/min. Absorbance was recorded at 214 nm. Preparative HPLC was performed on a Waters 600 system using a Vydac 218TP 22 × 250 mm column with a gradient of 0–40% B over 40 min at a flow rate of 16 mL/min. Mass spectra were acquired in positive ionisation mode on an API 2000 triple quadrupole mass spectrometer (SCIEX, Framingham, MA, USA). Protected Fmoc amino acids and O-(1H-6-chlorobenzotriazole-1-yl)-1,1,3,3-tetramethyluronium hexafluorophosphate (HCTU) were from Iris Biotech (Marktredwitz, Germany), *N*,*N*-dimethylformamide (DMF) and *N*,*N*-diisopropylethylamine (DIEA) were from Auspep (Melbourne, Australia). All other reagents were obtained from Sigma Aldrich.

### Simulation of the nirvana cabal

To evaluate the effect of venom/peptides on the behaviour and motor function of the zebrafish larvae, we analysed both the response of the animals to a manual mechanical touch response assay and their spontaneous swimming behaviour using the Zebrabox Revolution automatic tracking/screening platform (ViewPoint, France)^21^. Briefly, 5-dpf larvae and adult fish (500–600 mg) were exposed to dissected venom (0.001‒100 ng/μl), ρ-TIA and its analogues (0.001–10 μM), conantokins (0.003–1 μM) and conopressins (0.003–5 μM). The larvae and adults were tested in a 24-multiwell plate and a 0.5 L tank (Tecniplast, USA) respectively. The plates/tank were incubated in the Zebrabox Revolution and the animals were automatically tracked with a user-defined programme. Experiments were performed in light conditions and the swimming distance was defined as small (speed of 4 cm/s) and large (speed of 8 cm/s) distances. Zebrafish were monitored for a 5 min pre-treatment period and a 10 min post-treatment period, and data binned in 2 s intervals. Raw data and video were exported and processed *in silico* to export total swim distance for each group. At 15 min, treated fish were subjected to the touch response assay, as described below.

### Touch response assay

Larvae escape response was evaluated using a manual mechanical touch using a Drummond PCR mircopipet plunger (SDR scientific #5-000-1001-X10). Touch-evoked responses were scored 1–10 (1, absence of movement; 2, fin vibration without movement; 3, one body coil without movement; 4, several body coils with slight movement; 5‒9, gradual increment in swim distance; and 10 normal escape response with larvae reaching edge of the well). For each concentration, six larvae were tested individually, with each test comprising 10 separate manual stimulus scores. Untreated negative control responses (E3 medium only) were recorded in parallel using the same protocol.

### Systemic effects of conotoxins

Adult fish were administered 0.1 μg of dissected venom from each of the four venom duct sections by intramuscular (i.m.) injections (5–10 μL) using a Hamilton syringe (cemented point style 2, 26 s gauge needle) (Sigma-Aldrich no. 20795-U). Injected animals were placed in 1 L tanks under ambient light conditions, and monitored using the Zebrabox revolution (Viewpoint) imaging platform. Baseline swimming readings were obtained for 15 min before administering 5 μL of the dissected venom and the resulting behavior was recorded for further 15 min. Zebrafish were analysed for alterations in swimming pattern, and swimming distance relative to baseline quantified for each fish.

### Clone design and site-directed mutagenesis

The ZF α_1BB_-AR cDNA (NCBI Reference Sequence: NM_001007358.2) was designed and cloned into pcDNA3.1 (Genescript, USA), whereas the hamster α_1B_-AR cDNA was cloned into the pMT2’ vector (kind gift from Professor Bob Graham; Victor Chang Cardiac Research Institute, Sydney, Australia). The ZF α_1BB_-AR cDNA was subjected to *in vitro* site-directed mutagenesis to generate the double mutant E327D/S330F α_1BB_-AR cDNA, using specific primer pairs (Sigma Aldrich, Australia) and following the QuikChange mutagenesis kit (Stratagene). TOP10 *Escherichia coli* (Invitrogen) were transformed with WT or mutant cDNA and subsequently used for plasmid preparation using a PureLink® Quick Plasmid Miniprep Kit (Invitrogen) or High Speed Maxi kit (Qiagen). Purified cDNA was used to confirm all mutations by sequencing by the Australian Genome Research Facility.

### Transient expression of α_1_-ARs and membrane preparation

COS-1 cells (ATCC) were cultured in Dulbecco’s modified Eagle’s medium (DMEM, Invitrogen) containing glutamine and 5% fetal bovine serum. Cells were transiently transfected with purified plasmid DNA encoding WT or mutant α_1_-ARs using FuGENE HD (Promega) following the manufacturer’s protocol. 48 h post-transfection, cells were harvested and homogenized using a Polytron homogenizer (Brinkmann Instruments) in HEM buffer (20 mM HEPES, 1.5 mM EGTA, 12.5 mM MgCl_2_, pH 7.4) containing complete protease inhibitor (Roche Diagnostics). The homogenate was centrifuged at 484 × g (2000 rpm) for 10 min and the resulting supernatant was centrifuged at 23,665 × g (14000 rpm) for 30 min. The pellet was resuspended in HEM buffer containing 10% v/v glycerol and stored at −80°C prior to use. Protein concentration was determined using the BCA protein assay kit (Pierce) following the manufacturer’s protocol.

### Radioligand binding assay

The affinity of ρ-TIA at the α_1_-ARs was determined using the radiolabelled α_1_-AR antagonist ^3^H-prazosin (0.5 nM) and FlashBlue GPCR Scintillating Beads (PerkinElmer Life Sciences). Reactions containing radioligand, membranes from α_1_-AR-transfected COS-1 cells (5 µg protein), FlashBlue GPCR Scintillating Beads (100 µg/well) and increasing concentrations of ρ-TIA (100 pM–100 µM) in HEM buffer, were established in 96-well white polystyrene plates with clear flat bottoms in triplicate in a total reaction volume of 80 µl. After incubation for 60 min at room temperature with shaking, radioligand binding was detected using a Wallac 1450 MicroBeta scintillation counter (PerkinElmer Life Sciences).

### IP-one HTRF functional assay

COS-1 cells (ATCC) were cultured and transiently transfected with WT or mutant α_1_-AR DNA using FuGENE HD (Promega) following the manufacturer’s protocol. Assays measuring IP_1_-accumulation were performed 48 h post-transfection following the manufacturer’s instructions (IP-one HTRF assay kit, Cisbio international). In brief, the effect of increasing concentrations of norepinephrine (NE) (100 pM–100 µM), or ρ-TIA (100 pM–100 µM) were determined in the presence of 10 µM NE for WT and mutant ZF α_1BB_-AR or 1 µM NE for WT hamster α_1B_-AR. The assay was performed on 30,000 transfected cells in stimulation buffer in white 384 multiwell plates (Optiplate, PerkinElmer Life Sciences) incubated for 1 h at 37°C  with 5% CO_2_. Cells were then lysed by the addition of HTRF reagents before europium cryptate-labeled anti-IP_1_ antibody and the d2-labeled IP_1_ analog were added (IP-one HTRF assay kit, Cisbio International), followed by incubation for 1 h at room temperature. Emissions at 590 nm and 665 nm were recorded after excitation at 340 nm using a microplate reader (Tecan).

### Statistical analysis

Data were analysed using GraphPad Prism software, v7.0a (San Diego, USA). *In vivo* dose-response data was fitted to non-linear regression curves to establish EC_50_s. IP-one data was expressed as the HTRF ratio F = [(fluorescence_665nm_/fluorescence_590nm_) × 10^4^] normalized to the response elicited without ligand (negative control). For both the IP-one and the radioligand binding assay, non-linear regression curves were used to establish EC_50_ and IC_50_ values. All IC_50_ and EC_50_ values are expressed as the mean ± SEM averaged from six independent experiments, unless stated otherwise.

## Supplementary information

Supplementary Figure S1 and S2

Supplementary video S1
