# Supplementary material for: The α1-adrenoceptor inhibitor ρ-TIA facilitates net hunting in piscivorous Conus tulipa
Source: Sci Rep. 2019 Nov 28;9:17841. doi: 10.1038/s41598-019-54186-y (PMC6882899; doi:10.1038/s41598-019-54186-y)
Supplement: Supplementary file 1 — Supplementary Figure S1 and S2 [file 41598_2019_54186_MOESM1_ESM.pdf]

The α_1-adrenoceptor inhibitor ρ-TIA facilitates net hunting in piscivorous *Conus tulipa*

Mriga Dutt[1], Jean Giacomotto[2,3,4], Lotten Ragnarsson[1], Åsa Andersson[1], Andreas Brust[1], Zoltan Dekan[1], Paul F. Alewood[1] and Richard J. Lewis[1]*

[1] Centre for Pain Research, Institute for Molecular Bioscience, The University of Queensland, St. Lucia, QLD 4072, Australia; (MD) m.dutt@uq.edu.au; (LR) ; l.ragnarsson@imb.uq.edu.au;
(AR) a.andersson@imb.uq.edu.au; (AB) a.brust@imb.uq.edu.au;(ZD) z.dekan@imb.uq.edu.au;(PFA) p.alewood@imb.uq.edu.au

[2] Queensland Brain Institute, The University of Queensland, St. Lucia, QLD, 4072, Australia

[3] Queensland Centre for Mental Health Research, West Moreton Hospital and Health Service and University of Queensland, Brisbane, Australia; (JG) j.giacomotto@uq.edu.au

[4] Institut NeuroMyoGène, CNRS UMR5310, INSERM U1217, Université Claude Bernard Lyon 1, 69008 LYON

* Corresponding author: Richard J. Lewis, email: r.lewis@imb.uq.edu.au

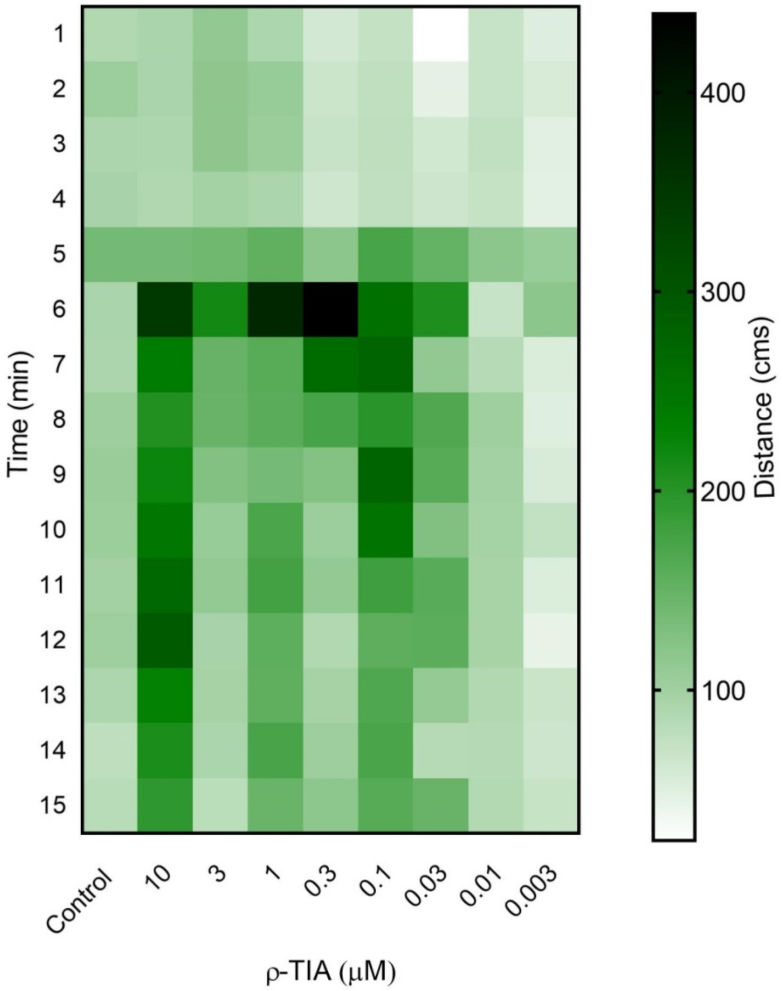

Supplementary Figure 1: ρ-TIA induced swimming burst on 5-dpf zebrafish larvae. Heat map plot showing the ρ-TIA induced swimming burst on larval motility. The swim distance of the larvae (right Y axis) has been plotted against time (left Y axis), and increase in swim distance is displayed by the gradient transitioning from white (smallest) to dark green (largest). Addition of the peptide at the 5th minute of the swim period induced an erratic, "burst" swimming behaviour that lasted ~ 1 min after which normal larval swimming was restored. The maximal burst response was observed at 0.3 µM concentration and this phenotype was absent at extremely low concentrations (0.01—0.003 µM) Untreated larvae did not produce this phenotype. Data has been presented as an average of six individual experiments.

```
                 173                                                        231
ZFA1ABADR        AAISVGPLFGWREPMPEDESVCRVNEDPGYAIFSAACSFYVPLAVILAMYCRVYVVARHK
ZFA1AAADR        ITISIGPLFGWKEPAPDDESICKITEEPGYAIFSALGSFYLPLVVILSMYCRVYVVARRE
HUMANA1AADR      LVISIGPLFGWRQPAPEDETICQINEEPGYVLFSALGSFYLPLAIILVMYCRVYVVAKRE
HAMSTERA1AADR    LVISIGPLFGWRQPAPEDETICQINEEPGYVLFSALGSFYVPLAIILVMYCRVYVVAKRE
RATA1AADR        LVISIGPLFGWRQPAPEDETICQINEEPGYVLFSALGSFYVPLAIILVMYCRVYVVAKRE
MOUSEA1AADR      LVISIGPLFGWRQQAPEDETICQINEEPGYVLFSALGSFYVPLTIILVMYCRVYVVAKRE
HUMANA1BADR      TVISIGPLLGWKEPAPNDDKECGVTEEPFYALFSSLGSFYIPLAVILVMYCRVYIVAKRT
HAMSTERA1BADR    TVISIGPLLGWKEPAPNDDKECGVTEEPFYALFSSLGSFYIPLAVILVMYCRVYIVAKRT
RATA1BADR        TVISIGPLLGWKEPAPNDDKECGVTEEPFYALFSSLGSFYIPLAVILVMYCRVYIVAKRT
MOUSEA1BADR      TVISIGPLLGWKEPAPNDDKECGVTEEPFYALFSSLGSFYIPLAVILVMYCRVYIVAKRT
ZFA1BAADR        FVISIGPLLGWKEPPSEDDTVCLITEEPFYALFSSLGSFYIPLAVILAMYCRVYIVAKRT
ZFA1BBADR        LVISIGPLLGWKEPPSPDDTVCAINEEPFYALFSSLGSFYIPLIVILVMYCRVYVVAKRT
                 232                                                          292
ZFA1ABADR        TRAMSKSRETNGL-SEQGMTLRIHCRKAQNDT-RKEDAMRLKNSHFAFMRLLKFSREKKA
ZFA1AAADR        TRGLISGQKTEKSDHAETVTLRIHRGNMTVSE-D----EALRNRTHFALRLLKFSREKKA
HUMANA1AADR      SRGLKSGLKTDKSDS-EQVTLRIHRKNAPAGG-SG--MASAKTKTHFSVRLLKFSREKKA
HAMSTERA1AADR    SRGLKSGLKTDKSDS-EQVTLRIHRKNVPAGG-GG--VNSAKNKTHFSVRLLKFSREKKA
RATA1AADR        SRGLKSGLKTDKSDS-EQVTLRIHRKNVPAEG-GG--VSSAKNKTHFSVRLLKFSREKKA
MOUSEA1AADR      SRGLKSGLKTDKSDS-EQVTLRIHRKNVPAEG-SG--VSSAKNKTHFSVRLLKFSREKKA
HUMANA1BADR      TKNLEAGVMKEMSNS-KELTLRIHSKNFHEDTLSSTKAKGHNPRSSIAVKLFKFSREKKA
HAMSTERA1BADR    TKNLEAGVMKEMSNS-KELTLRIHSKNFHEDTLSSTKAKGHNPRSSIAVKLFKFSREKKA
RATA1BADR        TKNLEAGVMKEMSNS-KELTLRIHSKNFHEDTLSSTKAKGHNPRSSIAVKLFKFSREKKA
MOUSEA1BADR      TKNLEAGVMKEMSNS-KELTLRIHSKNFHEDTLSSTKAKGHNPRSSIAVKLFKFSREKKA
ZFA1BAADR        TKNLEAGVMKERMDS-NELTLRIHYKGSQTQE-DCSK---GHLRSSLTVKLLKFSREKKA
ZFA1BBADR        TKNLEAGVKTESMNS-GEITLRIHRG-SQVHE-DAGKSRAHQARNSLTVKLLKFSREKKA
                 293                                                        352

ZFA1ABADR        AKTLGIVVGCFVLCWLPFFLVLPISSIFPSHRPPDAVFKITFWLGYFNSCLNPIIYPCFS
ZFA1AAADR        AKTLGIVVGCFVLCWLPFFLVLPIGSIFPTYRPSDTVFKITFWLGYFNSCINPIIYPCSS
HUMANA1AADR      AKTLGIVVGCFVLCWLPFFLVMPIGSFFPDFKPSETVFKIVFWLGYLNSCINPIIYPCSS
HAMSTERA1AADR    AKTLGIVVGCFVLCWLPFFLVMPIGSFFPDFKPSETVFKIVFWLGYLNSCINPIIYPCSS
RATA1AADR        AKTLGIVVGCFVLCWLPFFLVMPIGSFFPDFKPSETVFKIVFWLGYLNSCINPIIYPCSS
MOUSEA1AADR      AKTLGIVVGCFVLCWLPFFLVMPIGSFFPNFKPPETVFKIVFWLGYLNSCINPIIYPCSS
HUMANA1BADR      AKTLGIVVGMFILCWLPFFIALPLGSLFSTLKPPDAVFKVVFWLGYFNSCLNPIIYPCSS
HAMSTERA1BADR    AKTLGIVVGMFILCWLPFFIALPLGSLFSTLKPPDAVFKVVFWLGYFNSCLNPIIYPCSS
RATA1BADR        AKTLGIVVGMFILCWLPFFIALPLGSLFSTLKPPDAVFKVVFWLGYFNSCLNPIIYPCSS
MOUSEA1BADR      AKTLGIVVGMFILCWLPFFIALPLGSLFSTLKPPDAVFKVVFWLGYFNSCLNPIIYPCSS
ZFA1BAADR        AKTLGVVVGMFILCWLPFFLALPIGSFNTSLRPPETFFKVIFWLGYFNSCLNPIIYPCYS
ZFA1BBADR        AKTLGVVVGMFTLCWLPFFLTLPIVSFNTSLRPPETVSSIIFWLGYFNSCLNPIIYPCYS
```

Supplementary Figure 2: Binding pocket of ρ-TIA at the α_1-adrenergic receptors (AR). Multiple sequence alignment of the ρ-TIA binding pocket in the α_1-AR subtypes across mammals and zebrafish (duplicated forms; in red). ρ-TIA binds to the extracellular surface of the hamster α_{1B} subtype (in bold). The residues critical for binding of ρ-TIA are bolded and highlighted in yellow. Asp-327 and Phe-330 in the hamster α_{1B} adrenergic receptor are involved in ρ-TIA binding. Asp-327 forms a salt bridge with Arg-4 of TIA, and Phe-330 forms a cation-π interaction and T-stacking π interaction with Arg-4-TIA and Trp-3-TIA respectively [1]. Although a few residue modifications are observed in the zebrafish α_1-AR subtypes, the region around the binding pocket is highly conserved across species. The receptor sequences were retrieved from a BLAST similarity search and multiple sequence alignment was done on MUSCLE software. Gaps have been introduced to optimise the alignment.

Supplementary Video: ρ-TIA induces loss of escape response to touch stimulus in 5-dpf zebrafish larvae. Treatment with ρ-TIA reduced the escape response of the larvae to mechanical touch stimuli in a concentration-dependant manner. Top panel (from left to right): untreated control (E3), 10 µM TIA and 3 µM TIA. Bottom panel (from left to right): 1 µM TIA, 0.3 µM TIA and 0.1 µM TIA.

References

1 Ragnarsson, L. *et al.* Conopeptide ρ-TIA defines a new allosteric site on the extracellular surface of the α1B-adrenoceptor. *J Biol Chem* **288**, 1814-1827, doi:https://doi.org/10.1074/jbc.M112.430785 (2013).
